# PPARγ Expression Is Diminished in Macrophages of Recurrent Miscarriage Placentas

**DOI:** 10.3390/ijms19071872

**Published:** 2018-06-26

**Authors:** Theresa Maria Kolben, Elisabeth Rogatsch, Aurelia Vattai, Anna Hester, Christina Kuhn, Elisa Schmoeckel, Sven Mahner, Udo Jeschke, Thomas Kolben

**Affiliations:** 1 Department of Obstetrics and Gynecology, University Hospital, LMU Munich, Marchioninistr. 15, 81377 Munich, Germany; Theresa.kolben@med.uni-muenchen.de (T.M.K.); elisabeth.rogatsch@icloud.com (E.R.); Aurelia.vattai@med.uni-muenchen.de (A.V.); anna.hester@med.uni-muenchen.de (A.H.); Christina.kuhn@med.uni-muenchen.de (C.K.); sven.mahner@med.uni-muenchen.de (S.M.); Thomas.kolben@med.uni-muenchen.de (T.K.); 2 Department of Pathology, LMU Munich, Marchioninistr. 27, 81377 Munich, Germany; elisa.schmoeckel@med.uni-muenchen.de

**Keywords:** PPARγ, first trimester placenta, decidual macrophages, miscarriage

## Abstract

PPARγ belongs to the group of nuclear receptors which is expressed in the trophoblast and together with other factors is responsible for the maintenance of pregnancy. Apart from that PPARγ is also a main factor for macrophage polarization. The aim of this study was to investigate the combined expression pattern and frequency of PPARγ under physiological circumstances and in spontaneous and recurrent miscarriages in the trophoblast and in maternal macrophages of the decidua. Human placental tissues of the first trimester (15 physiologic pregnancies, 15 spontaneous abortion and 16 recurrent miscarriage placentas) were analyzed for expression of the nuclear receptor PPARγ. Expression changes were evaluated by immunohistochemistry and real time PCR (RT-PCR) in trophoblast and in maternal macrophages of the decidua. Maternal macrophages were identified by double immunofluorescence using cluster of differentiation 68 (CD68) as marker for macrophages and further characterized regarding their M1/M2 polarization status. The intermediate villous trophoblast revealed a significantly lower PPARγ expression in spontaneous and recurrent abortion. Maternal macrophages express PPARγ. Their number is significantly enhanced in the decidua of spontaneous miscarriages whereas in recurrent miscarriages maternal macrophages seem to express PPARγ only in very few cases. PPARγ is associated with an M2 polarization state that is common for decidual macrophages. The lack of PPARγ in recurrent miscarriage decidual macrophages seems to be associated with a specific inflammatory response against the fetus.

## 1. Introduction

Miscarriage, which is defined as either spontaneous or recurrent, is a common disorder in pregnancy [1]. It affects 25–50% of all reproductive-aged women. Immunologic, endocrine and metabolic mechanisms are involved in the success of human pregnancy and disturbances in any of these processes can lead to fetal loss [2]. Established risk factors are fetal chromosomal or endocrine disorders for spontaneous miscarriages and the antiphospholipid syndrome, thrombophilia or maternal anatomical malformations, especially for recurrent pregnancy losses [3]. In nearly 50% of affected patients, however, the cause of miscarriage remains unknown [4].

Peroxisome proliferator-activated receptor γ (PPARγ) belongs to the family of nuclear receptors [5] that are key players in maintaining pregnancy [6,7]. PPARγ, together with its heterodimer binding partner retinoid X receptor alpha (RXRα), are involved in cell proliferation, cell differentiation, and organogenesis [8]. RXRα is upregulated in extravillous trophoblast in recurrent miscarriages in humans [9]. RXRα plays a pivotal role in the receptor family, due to its ability to form heterodimers with other nuclear receptors. Heterodimer partners include, e.g., peroxisome proliferator-activated receptor (PPAR), thyroid hormone receptor (TR), and liver X receptor (LXR) [2,10,11,12]. Especially the expression of the isoform PPARγ is linked to trophoblast invasion [13] and downregulation of the isoform RXRα seems to protect from apoptosis in human trophoblasts [9].

Not only trophoblast cells express PPARγ, but also macrophages [14]. Macrophages play a key role in immune response and they can respond to environmental stimuli by acquiring specific phenotypes [15]. In response to external cues they will undergo classical M1 activation with high levels of inflammation and microbicide as well as anti-tumor activity. Alternatively, the M2 pathway contains mostly parasite containment, tissue remodeling and most importantly in this case immunomodulatory functions like pregnancy [16,17,18].

Our former studies showed that the number of decidual macrophages is increased at the feto-maternal interface of preeclampsia placentas [19] and also in spontaneous miscarriage cases. An additional finding was the FasL-positivity of these macrophages [20]. Therefore, the aim of this study was a phenotype characterization of macrophage populations in abortive placental tissue, its PPARγ expression and the characterization of PPARγ expressing trophoblast sub-types.

## 2. Results

### 2.1. Immunohistochemistry

#### 2.1.1. PPARγ-Expression in the Trophoblast

**Figure 1:**
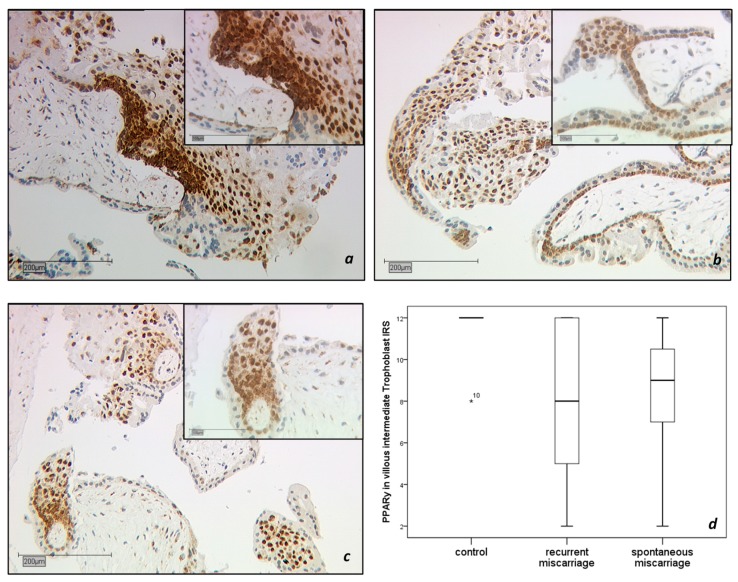
Immunohistochemical staining of Peroxisome proliferator-activated receptor gamma (PPARγ) in the villous trophoblast. PPARγ expression is found with high distribution and intensity in intermediate villous trophoblastic cells (IVT) of first-trimester placentas. The control group (15 cases) showed the strongest expression pattern (**a**). PPARγ was significantly downregulated (*p* = 0.01) in trophoblastic tissue of recurrent miscarriage (RM, 16 cases, (**b**). PPARγ expression in the IVT of spontaneous miscarriage tissue (SM, 15 cases), (**c**) was significantly decreased compared to the control (*p* = 0.001). The boxplot summarizes the statistical data of the immunohistochemical staining results (**d**). Scale is 200 μm. The insert picture is 100 μm scaled.

The expression of PPARγ in the nucleus and cytoplasm of trophoblast cells was analyzed in tissue from healthy pregnancies (15 cases), spontaneous miscarriages (SM, 15 cases), and recurrent miscarriages (RM, 16 cases; Figure 1a–d). The intermediate villous trophoblast (IVT) revealed a significantly lower expression in the group with recurrent abortions (Figure 1b) than in the group with healthy placentas (Figure 1a, IRS 8 vs. 12, *p* = 0.01). There was a significant downregulation of PPARγ in the IVT of spontaneous abortions (Figure 1c, IRS 9 vs. 12, *p* = 0.001) compared to the control group. Briefly, the staining results are shown in the boxplot in Figure 1d.

#### 2.1.2. CD68 Positive Decidual Macrophages in the Decidua

**Figure 2:**
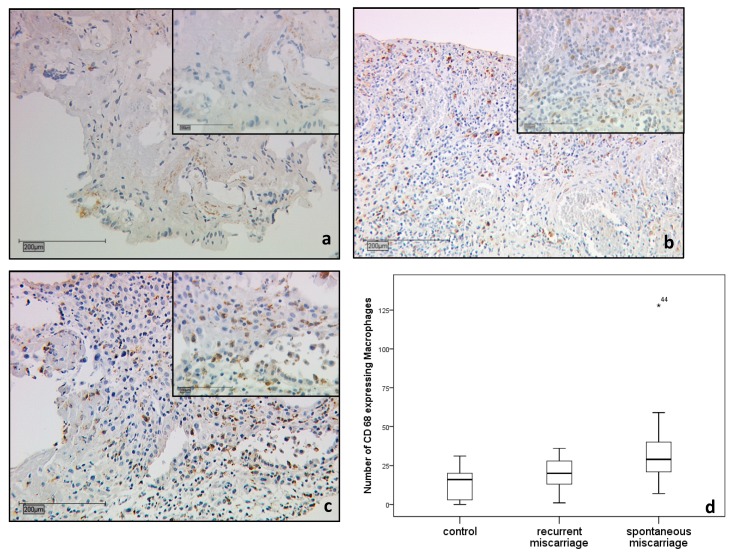
Immunohistochemical staining of decidual macrophages with CD68 as a marker for macrophage positivity. Decidual macrophages were increased in RM (16 cases) and SM samples (15 cases) compared to the control group (15 cases), (**a**). In recurrent miscarriage specimens, the decidual macrophages tended to be upregulated (*p* = 0.181) (**b**). In spontaneous miscarriage samples, the population of macrophages was significantly higher compared to the control (*p* = 0.013) (**c**). Summary of staining results of CD68 positive decidual macrophages (**d**). Scale is 200 μm. The insert picture is 100 μm scaled.

CD68 positive macrophages were investigated in the placenta of healthy pregnancies (15 cases), SM (15 cases), and RM (16 cases; Figure 2a–d). The number of CD68 positive macrophages was low in the decidua basalis of control specimens (Figure 2a). The macrophages were slightly increased in decidua basalis RM samples, but without statistical significance (*p* = 0.181) (Figure 2b; median number of macrophages = 21 vs. 16). Decidual macrophages were significantly increased in the decidua basalis SM group (*p* = 0.013) (Figure 2c; median number of macrophages 32 vs. 16). A summary of the staining results is shown in Figure 2d.

### 2.2. Double Immunofluorescence

#### Identification of PPARγ-Expressing Cells in the Decidua Basalis

**Figure 3:**
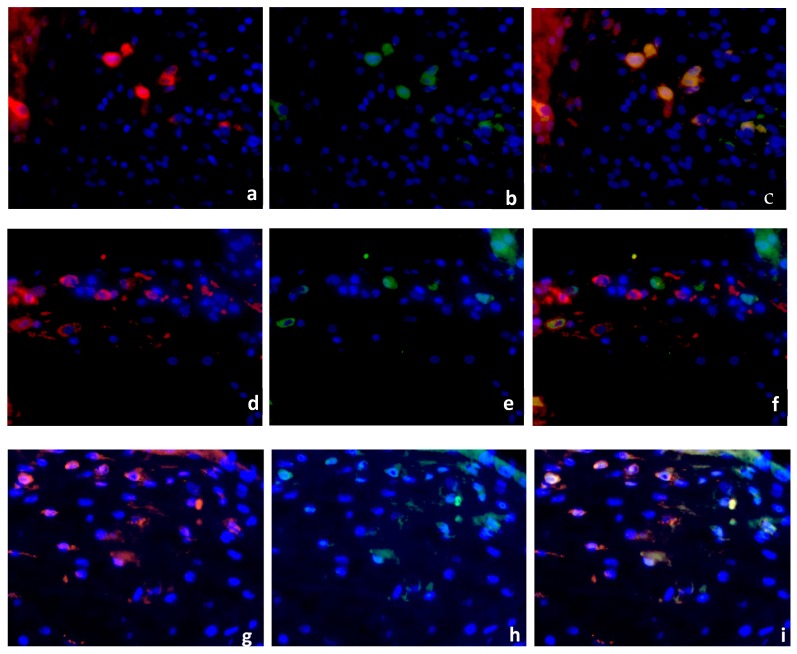
Double Immunofluorescence of CD68 & PPARγ. CD68 (red staining), PPARγ (green staining), nuclear staining (blue). CD68 (**a**) and PPARγ (**b**) are expressed in the decidua of healthy placenta (15 cases), co-expression presented as triple filter yellow (**c**). High distribution of CD68 positive macrophages was found in RM (16 cases), (**d**). PPARγ-positive cells are shown in (**e**). Triple filter excitation showed an absence of PPARγ positive macrophages (**f**). Both markers (CD68, (**g**) and PPARγ, (**h**)) are co-expressed in the group of SM (15 cases), (**i**). All pictures are 40× lens.

Decidua basalis tissue of regular first trimester pregnancies (15 cases), SM (15 cases) and RM (16 cases) was double stained using antibodies against PPARγ (green staining), and CD68 (red staining). Nuclear staining appeared in blue. PPARγ + CD68 double immunofluorescence staining was performed to investigate the macrophage expression of PPARγ in RM, SM and control groups. CD68 staining in the cytoplasm of normal decidual cells is shown in Figure 3a. Figure 3b presents the cytoplasmic staining of PPARγ positive cells from the same area. The depiction of CD68 and PPARγ is represented as a co-expression by triple filter excitation in Figure 3c. Both markers are ubiquitously expressed in the healthy placenta (Figure 3a–c). A large number of CD68-positive macrophages was observed in RM samples (Figure 3d), although with almost no PPARγ expression (Figure 3e). Triple filter excitation demonstrates (Figure 3f) a near absence of PPARγ in macrophages of recurrent miscarriages. A large population of CD68 positive macrophages (Figure 3g) and PPARγ expressing cells (Figure 3h) was detected in SM samples. Figure 3i shows a strong co-expression of both markers. We identified CD68 positive macrophages also expressing PPARγ in the healthy and in the spontaneous miscarriage placenta. In the group of recurrent miscarriages only very few PPARγ-expressing macrophages (5–8% of the macrophages in RM are PPARγ-positive) were detected.

### 2.3. Characterization of the Macrophage Population in Recurrent Miscarriage Cases

**Figure 4:**
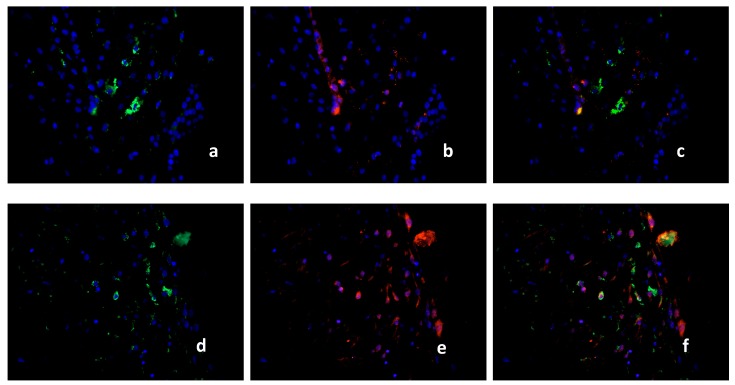
Double Immunofluorescence of CD68 and iNOS. CD68 (green staining), iNOS (red staining), nuclear staining (blue). CD68 (**a**) and iNOS (**b**) are co-expressed only in few macrophages in the decidua of healthy placenta, presented as triple filter yellow (**c**). High distribution of CD68 positive macrophages was found in RM (**d**). iNOS positive cells are shown in (**e**). Triple filter excitation shows a co-expression of iNOS and macrophages (**f**). All pictures are 40× lens.

**Figure 5:**
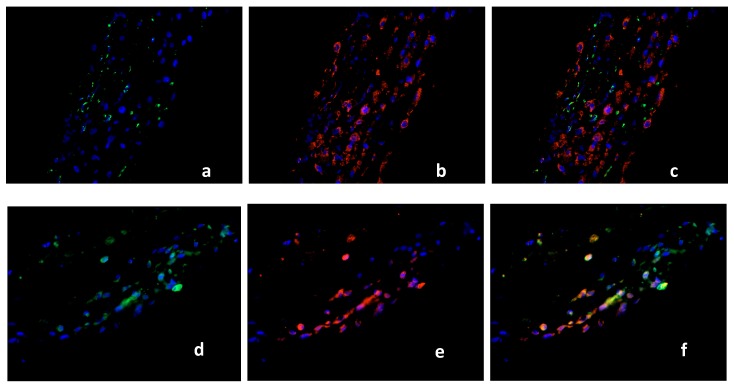
Double Immunofluorescence of CD68 and TLR2. CD68 (green staining), TLR2 showed strong expression in the healthy decidua (red staining), nuclear staining (blue). CD68 (**a**) and TLR2 (**b**) are not co-expressed in the decidua of healthy placenta, presented as triple filter yellow (**c**). CD68 positive macrophages in RM placenta (**d**). TLR2 positive cells are shown in (**e**). Triple filter excitation shows a co-expression of TLR2 and macrophages (**f**) in the RM placenta. All pictures are 40× lens.

**Figure 6:**
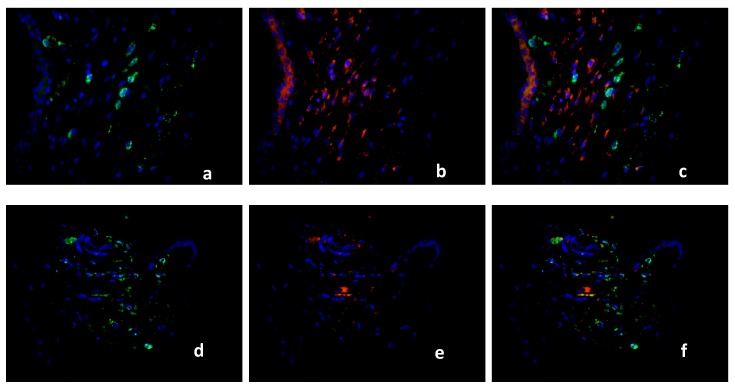
Double Immunofluorescence of CD68 and CCL1. CD68 (green staining), CCL1 showed intense expression in the healthy decidua (red staining), nuclear staining (blue). CD68 (**a**) and CCL1 (**b**) are co-expressed in the decidua of healthy placenta in a number of cells, presented as triple filter yellow (**c**). CD68 positive macrophages in RM placenta (**d**). Only few CCL1 positive cells are shown in (**e**). Triple filter excitation shows a diminished co-expression of CCL1 and macrophages (**f**) in the RM placenta. All pictures are 40× lens.

**Figure 7:**
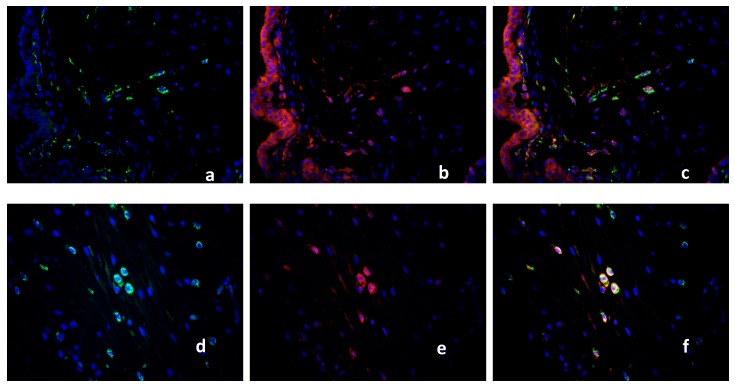
Double Immunofluorescence of CD68 and CX3CR1. CD68 (green staining), CX3CR1 showed expression in a variety of different cell types including endometrial glands and decidual stromal cells [21] in the healthy decidua (red staining), nuclear staining (blue). CD68 (**a**) and CX3CR1 (**b**) are co-expressed in the decidua of healthy placenta in almost all CD 68-positive cells (>95%), presented as triple filter yellow (**c**). CD68 positive macrophages in RM placenta (**d**). Only 40–50% CX3CR1 expressing cells (**e**) showed co-expression with CD68, as shown in (**f**). All pictures are 40× lens.

Because macrophages in RM cases (16 cases) expressed PPARγ only in 5–8% of the total macrophage population compared to healthy control tissue (15 cases) and SM (15 cases), we further characterized these cells with a panel of M1/M2 markers and CD68. The M1 marker iNOS is expressed in 2–5% of the macrophages in healthy controls (CD 68 Figure 4a; iNOS Figure 4b, triple filter excitation Figure 4c). In RM cases, iNOS is expressed in 90% of the macrophages (CD68 Figure 4d, iNOS Figure 4e, triple filter excitation Figure 4f). TLR2 is intensely expressed in the healthy decidua but showed no co-expression with macrophages (CD 68 Figure 5a, TLR2 Figure 5b, triple filter excitation Figure 5c). In RM cases, TLR2 as M1 marker is co-expressed with macrophages in 80–90% of the cases (CD68 Figure 5d, TLR2 Figure 5e, triple filter excitation Figure 5f). The chemokine CCL1 is a marker for M2b polarized macrophages. In healthy controls, 40–50% of the macrophages showed co-expression with CCL1 (CD68 Figure 6a, CCL1 Figure 6b, triple filter excitation Figure 6c). In RM cases, only 5–10% of the macrophages showed co-expression with CCL1 (CD68 Figure 6d, CCL1 Figure 6e, triple filter excitation Figure 6f). CX3C chemokine receptor 1 (CX3CR1) as a M2 macrophage marker is widely expressed (>95%) on healthy control decidual macrophages (CD68 Figure 7a, CX3CR1 Figure 7b, triple filter excitation Figure 7c). In RM cases, only 40–50% of the macrophages showed co-expression with C X3CR1 (CD68 Figure 7d, CX3CR1 Figure 7e, triple filter excitation Figure 7f).

### 2.4. Evaluation of PPARγ Expression with Real-Time RT-PCR (TaqMan)

**Figure 8:**
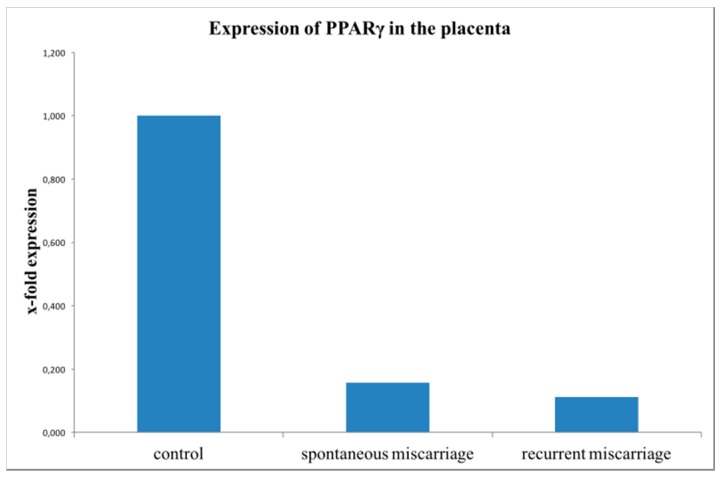
Results of PPARγ mRNA expression analysis with TaqMan RT-PCR from trophoblastic tissue. PPARγ mRNA expression was significantly downregulated in the miscarriage groups (SM, 15 cases, *p* = 0.01) and RM, 16 cases, *p* = 0.004)) compared to the healthy controls (15 cases). This bar graph shows the mean of relative PPARγ expression; therefore, the presentation of error bars is not appropriate.

PPARγ mRNA (PPARG) expression was analyzed in placental tissue from SM, RM and healthy controls by quantitative RT-PCR. PPARγ was significantly downregulated in SM (15 cases, 1.8-fold; *p* = 0.010) and in RM (16 cases, 1.5-fold; *p* = 0.004) compared to the control group (15 cases, Figure 8).

## 3. Discussion

Within this study we could show that PPARγ is downregulated in the intermediate villous trophoblast (IVT) in both spontaneous (SM) and recurrent miscarriage (RM) placentas. The downregulation of PPARγ was confirmed by RT-PCR in both miscarriage pregnancy cases.

In addition, we showed that in recurrent miscarriages, decidua basalis macrophages are nearly PPARγ-negative, whereas in normal controls and surprisingly also in SM decidua basalis macrophages are all PPARγ-positive. The additional characterization of the macrophage polarization status using the M1 polarization markers TLR2 and iNOS [22] and the M2 polarization markers CCL1 and CX3CR1 confirmed the loss of M2 polarized marcrophages [23] in recurrent miscarriages.

PPARγ as nuclear receptor is already known to be essential for the maturation of alternatively activated M2 macrophages [24]. M2 macrophages and decidual macrophages have mainly immune regulatory and homeostatic properties [25]. These macrophages have little in common with pro-inflammatory M1 macrophages, which is in line with the role for decidual macrophages in establishing and sustaining fetal tolerance [26].

In addition, rosiglitazone as a selective peroxisome PPARγ agonist has been shown to induce an M2 macrophage polarization via activating the PPARγ pathway [27,28]. The activation of PPARγ suppresses gene transcription by interfering with signal transduction pathways, such as the nuclear factor 'kappa-light-chain-enhancer' of activated B-cells (NF-κB), Signal transducer and activator of transcription (STAT), and Activator protein 1 (AP-1) pathways that are involved in pro-inflammatory immune responses [29,30,31]. It is a striking result of this study, that we could identify the loss of PPARγ in decidual macrophages of patients with recurrent miscarriages but not in patients with SM and of course in normal control placentas.

In spontaneous miscarriages, we identified a significant increase of decidual macrophages, although they were PPARγ positive. In former studies, we could show that these macrophages express FasL [20]. The expression of FasL on decidual macrophages had been already described before [32].

The role of the Fas/FasL system in the conditions of spontaneous abortion and pregnancy had been described for T cell apoptosis by decidual and trophoblast cells earlier [33,34]. Our group was able to describe an increased expression of FasL in decidual macrophages of spontaneous miscarriages [20]. Therefore, we speculated that FasL expression by macrophages could be a part of an M2-like polarization. FasL-expressing macrophages could induce apoptosis to Fas-bearing activated T-cells reducing potentially harmful immune responses against the semi-allogenic embryo. We further assumed that macrophages might mediate the T-cell triggered trophoblast apoptosis highlighting an alternative way of inducing apoptosis in SM [15].

Placental growth is exponential in the first trimester of pregnancy and involves coordinated events in trophoblast and mesenchyme, one of these is the differentiation of progenitor cytotrophoblast cells into intermediate villous trophoblast cells (IVT). These IVT are programmed to either fuse with the syncytium, or are transferred to extravillous trophoblast cells [35]. We found a downregulation of PPARγ in the IVT compartment in both spontaneous and recurrent miscarriage on protein as well as on mRNA-level. Already decades ago, the natural binding partner of PPARγ, the RXRα was described in this trophoblast compartment: RAR and RXR, both types of receptors were present in the proliferative intermediate villous trophoblast [36]. Later, RXRα was found to play a crucial role in pregnancy and is a key regulator of apoptosis in trophoblasts of patients with recurrent miscarriages [9]. PPARγ, on the other hand, was also described to be dysregulated in different trophoblast compartments of the miscarriage placenta [37,38], although PPARγ expression in the IVT was never investigated before. Interestingly, Fournier et al. described that activation of PPARγ induces accumulation of lipids, villous trophoblast differentiation and inhibits trophoblast invasiveness [39]. In addition, the expression of PPARγ is downregulated by stimulation of trophoblast cells with either arachidonic acid or 15d-PGJ2 [40]. Because we identified a downregulation of PPARγ in the IVT of miscarriages and a missing PPARγ expression in macrophages of RM cases, we might speculate that PPARγ ligands (e.g., prostaglandins) are released to a higher extent under these pathological circumstances.

## 4. Materials and Methods

### 4.1. Patient Data

The Institutional Review Board of the Ludwig-Maximilian-University, Munich, (Number of approval: 337-06, 29 December 2006) approved this study. All women signed an informed consent allowing analysis of all clinical and laboratory data mentioned in this study. Placental tissue from spontaneous miscarriages (SM) (*n* = 15) and recurrent miscarriages (RM) (*n* = 16) at gestational weeks 4 to 13 was obtained at the Department of Obstetrics and Gynecology, LMU Munich. Placental tissue from legal terminations of healthy pregnancies (*n* = 15) served as control group. The tissue was collected at a private practice clinic in Munich, Germany. The control group specimens were confirmed as healthy by a blinded independent pathologist. All placental material was acquired by dilatation and curettage, without any prior pharmaceutical induction. In cases of SM and RM, the operation was performed within 24 h after diagnosis. Instantly, after the uterine curettage, the obtained tissue was either frozen or formalin fixed for further analysis. All patients included had an inconspicuous family and medical history, which was obtained systematically. Patients with common disorders, autoimmune diseases, thrombophilia and microbiological infections (Bacteria and Chlamydia trachomatis) were excluded. Chromosomal abnormalities were ruled out by karyotype analysis in all samples, as described recently [11,41]. Table 1 summarizes the number of samples used for immunohistochemical staining for each gestational week. Table 2 shows the demographic and clinical characteristics of the study population.

### 4.2. Immunohistochemistry

Formalin-fixed tissue slides were embedded in paraffin wax for immunohistochemistry. Samples were deparaffinized in xylol for 20 min and rinsed in 100% ethanol. Methanol/H_2_O_2_ incubation for 20 min was performed to inhibit endogenous peroxidase reaction. Afterwards, the specimens were rehydrated in deescalating alcohol gradients, starting with 100% ethanol and ending with distilled water. The samples were cooked in a pressure pot, containing a sodium citrate buffer (pH = 6.0), which consisted of 0.1 mM citric acid and 0.1 mM sodium citrate in distilled water. Subsequently, samples were washed in PBS twice and incubated with a blocking solution (reagent 1, ZytoChem Plus HRP Polymer System (Mouse/Rabbit), Zytomed, Berlin, Germany) for 5 min. Incubation with the primary antibody was performed with each section for 16 h at 4 °C. All antibodies used are listed in Table 3. Following every subsequent step, samples were washed twice in PBS (pH = 7.4). Blocking solutions, containing post block (reagent 2) for 20 min and HRP-Polymer (reagent 3) for 30 min, were applied. The chromogen-substrate staining was carried out using the Liquid DAB+ Substrate Chromogen System (Dako Scientific, Glostrup, Denmark), 1 min for CD68 and 2 min for PPARγ. The reaction was stopped by applying distilled water. Finally, tissue samples were counterstained with Hemalaun for 2 min and blued in tap water. Specimens were dehydrated in an ascending alcohol gradient and cover slipped with Eukitt^®^ quick hardening mounting medium (Sigma Aldrich, St. Louis, MO, USA). Positive control (human colon tissue) as well as negative control staining was carried out as described previously [10,40]. All slides were analyzed using the microscope Leitz Wetzlar (Wetzlar, Germany; Type 307-148.001 514686). The immunoreactive score (IRS) was used for evaluation of the intensity and distribution pattern of antigen expression. This semi-quantitative score is calculated as follows: the optical staining intensity (grades: 0 = none, 1 = weak, 2 = moderate, 3 = strong staining) is multiplied by the total percentage of positively stained cells (0 = none, 1 ≤ 10%, 2 = 11–50%, 3 = 51–80% and 4 ≥ 81% of the cells). This multiplication has a minimum of 0 and a maximum of 12. Analysis of all slides was performed independently by two experienced staff members. Total number of macrophages in a magnification field of 40× lens was calculated three times each in 3 different areas of the decidua basalis. The median number was calculated.

### 4.3. Immunofluorescence

#### 4.3.1. Evaluation of PPARγ-Expressing Cells as Macrophages

For the visualization of PPARγ-expressing cells in the trophoblast, tissue samples of SM, RM, both first-trimester abortion placentas, and healthy controls (first trimester) were used. The antibodies used are shown in Table 3. Double immunofluorescence staining for PPARγ and CD68 as a specific macrophage marker was performed to identify expression patterns in the nucleus and the cytoplasm.

#### 4.3.2. Evaluation of M1/M2 Marker on Decidual Macrophages

In order to further characterize the macrophage polarization state, TLR2 and iNOS, as well as CCL1 and CX3CR1, were used for M1 and M2 polarization, respectively. Each specimen was incubated overnight at 4 °C with monoclonal anti-CD68 mouse IgG1 and one of the polyclonal IgG antibodies against PPARγ, TLR2, iNOS, CCL1, or CX3CR1. Polyclonal Cy-2- and polyclonal Cy-3-conjugated antibodies (Dianova, Hamburg, Germany) were used as secondary antibodies. Incubation was performed for 30 min at room temperature. Samples were fixed with Vectashield^®^ mounting medium with DAPI (Vector Laboratories; Burlingame, CA, USA) and analyzed with the Axioskop fluorescent photomicroscope (Zeiss; Oberkochen, Germany). Images were taken with the Axiocam camera system (Zeiss CF20DXC).

### 4.4. Evaluation of PPARγ with Real-Time RT-PCR (Taq Man)

#### 4.4.1. RNA Extraction from Placental Tissue

mRNA extraction was accomplished using the placental tissue of 15 women with SM, 16 women with RM and 15 healthy controls from the 7th to the 12th week of gestation. RNA extraction using 10 mg tissue of each sample was accomplished with RNeasy^®^ Lipid Tissue Mini Kit (Qiagen, Hilden, Germany) according to the manufacturer’s protocol.

#### 4.4.2. Reverse Transcription

According to the protocol reverse transcription (RT) was carried out with the High Capacity cDNA Reverse Transcription Kit (Applied Biosystems™, Fisher Scientific Company, Waltham, MA, USA) and placed in a mastercycler^®^ gradient (Eppendorf, Hamburg, Germany). RT conditions were as follows: 10 min at 25 °C, 2 h at 37 °C, 5 min at 85 °C and continued by a hold step at −20 °C.

#### 4.4.3. Real-Time Reverse Transcription PCR

After conversion of RNA to cDNA, PCR was performed on all samples individually. Real-Time Reverse Transcription PCRs were covered with optical caps in optical 96-well (Applied Biosystems™, Fisher Scientific Company, Waltham, MA, USA) reaction microtiter plates. Each reaction was accomplished with a volume of 20 µL, including 1 µL cDNA, 8 µL H_2_O (DEPC treated DI water; Sigma, Taufkirchen, Germany) and 10 µL TaqMan^®^ Fast Universal PCR Master Mix 2× (Applied Biosystems, Nr. 4367846; 50 mL). The total contained 1 µL TaqMan^®^ Gene Expression Assay 20× (HS01115513_m1 for PPARγ, Applied Biosystems). The temperature protocol was as follows: 20 s at 95 °C, 40 cycles of amplification, denaturation for 3 s at 95 °C and denaturation plus annealing process for 30 s at 60 °C. Processing the PCR assays was performed using the 7500 Fast Real-Time PCR System (Applied Biosystems), and quantification was accomplished by the 2^−ΔΔ*C*t^ method using β-actin as housekeeping gene (Applied Biosystems, Hs_99999903_m1).

### 4.5. Statistics

Analysis of the collection and statistical data was processed with the SPSS software version 24 (SPSS, Chicago, IL, USA) and Excel version 12.3.1 (Microsoft Windows 2016; Redmond, WA, USA). The Mann-Whitney U signed-rank test was used for the comparison of two independent groups. *p*-values < 0.05 were considered to be statistically significant.

## 5. Conclusions

Mouse knockout models showed that PPARγ is essential for placentation. PPARγ depletion leads to fetal loss in early pregnancy due to the missing PPARγ expression and extended placental defects [42]. In addition, decidual M1-like macrophage polarization events are associated with PPARγ modulation strategies [14]. Therefore, the PPARγ pathway is a new molecular target for future preventive strategies for the treatment of spontaneous and recurrent miscarriages.

## Figures and Tables

**Table 1:** Number of slides used for immunohistochemical staining for each gestational week.

Gestational Age	Normal Pregnancy	Spontaneous Miscarriage	Recurrent Miscarriage
4th week	0	0	1
7th week	2	1	3
8th week	4	5	5
9th week	2	2	4
10th week	3	3	0
11th week	0	3	2
12th week	3	1	1
13th week	1	0	0
	*n* = 15	*n* = 15	*n* = 16

**Table 2:** Demographic and clinical characteristics of the study population.

Characteristics *	Normal Pregnancy *n* = 15	Spontaneous Miscarriage *n*= 15	Recurrent Miscarriage *n*= 16	*p* Value (Kruskal Wallis Test)
Maternal age (years)	31.18 ± 8.06 (18.7−43.3)	37.8 ± 4.51 (29.2–43.2)	35.76 ± 4.8 (29.5–46.9)	0.049
Gestational age (weeks)	9.53 ± 1.95 (7–13)	8.4 ± 1.89 (7–12)	9.3 ± 1.49 (4–12)	0.276
Gravidity	4 ± 1.8 (1–7)	1.6 ± 0.9 (1–4)	3.1 ± 1.1 (2–5)	0.001
Parity	2 ± 1.1 (0–4)	0.9 ± 0.8 (0–2)	0.3 ± 0.6 (0–2)	0.003

Values are Mean ± S.D. * Mean, standard deviation, range.

**Table 3:** Antibodies used for immunohistochemical characterization and double immunofluorescence of placental tissue samples.

Antibody	Isotype	Clone	Dilution	Source
PPARγ ^a,b^	rabbit IgG	polyclonal	1:500 in PBS ^a^ 1:500 in Dako ^b^	Abcam Serotec, Cambridge, UK DAKO (S322); Carpenteira, CA, USA
CD 68 ^a,b^	mouse IgG1	monoclonal	1:8000 in PBS ^a^ 1:8000 in Dako ^b^	Sigma Aldrich (CL1346), St. Louis, MO, USA DAKO (S322); Carpenteira, CA, USA
iNOS ^b^	Rabbit IgG	polyclonal	1:3000 in Dako	Thermo Scientific, (NPA3-030A) DAKO (S322); Carpenteira, CA, USA
TLR2 ^b^	Rabbit IgG	polyclonal	1:750 in Dako	Sigma Aldrich, St. Louis, MO, USA Dako (S322); Carpenteira, CA, USA
CCL1 ^b^	Rabbit IgG	polyclonal	1: 50 in Dako	Sigma Aldrich, St. Louis, , MO, USA Dako (S322); Carpenteira, CA, USA
CX3CR1 ^b^	Rabbit IgG	polyclonal	1: 400 in Dako	Abcam Serotec, Cambridge, UK Dako (S322); Carpenteira, CA, USA
Cy-2 or -3 ^b^	goat IgG anti-mouse	polyclonal	1:500 ^b^	Dianova, Hamburg, Germany
Cy-2 or -3 ^b^	goat IgG anti-rabbit	polyclonal	1:100 ^b^	Dianova, Hamburg, Germany

^a^ antibodies used for immunohistochemistry, ^b^ antibodies used for immunofluorescence.

## Abbreviations

MDPI	Multidisciplinary Digital Publishing Institute
PPAR	Peroxisome-Proliferator-activated Receptor
SM	Spontaneous miscarriage
RM	Recurrent miscarriage
